# A Substrate Integrated Waveguide-Based W-Band Antenna for Microwave Power Transmission

**DOI:** 10.3390/mi13070986

**Published:** 2022-06-24

**Authors:** Liang Liu, Yu Yang, Chuan Yu, Shifeng Li, Hao Wu, Limin Sun, Fanbao Meng

**Affiliations:** Science and Technology on High Power Microwave Laboratory, Institute of Applied Electronics, China Academy of Engineering Physics, Mianyang 621900, China; liu941205@163.com (L.L.); yuyanghd@gmail.com (Y.Y.); lishifeng@alu.uestc.edu.cn (S.L.); wuhao@ieee.org (H.W.); slm14@163.com (L.S.); fanbao_meng@126.com (F.M.)

**Keywords:** antenna, W-band, substrate integrated waveguide (SIW), coplanar waveguide (CPW)

## Abstract

A W-band slot array antenna based on a substrate integrated waveguide (SIW) for microwave power transmission (MPT) is proposed in this paper. By size optimization, the transition from the rectifier element to the antenna is limited to a small size. It realizes a compact arrangement of the radiating slots, which not only improves the aperture efficiency of the antenna but also makes it easy to integrate into a large-scale array. For antenna testing, a coplanar waveguide–SIW–rectangular waveguide transition structure is added at the end of the antenna, and an antenna with this transition structure is processed by PCB printing technology and measured. The measured reflection coefficient is less than −10 dB at 90–96 GHz, the aperture efficiency is greater than 60% at 93.5–94.5 GHz, the maximum gain is 13.2 dB at 93.5 GHz, and the aperture efficiency is 79%. The test results of the antenna show that the antenna has a good performance and can be applied to the MPT system as a rectenna.

## 1. Introduction

Microwave power transmission (MPT) [1,2,3] refers to the transmission of energy directly from the transmitter to the receiver through free space, and its transmission loss is only caused by atmospheric loss, rain attenuation, shielding, etc. Microwave beam intensity and direction are easy to control, a quality which has gained more and more attention. The rectenna, as a key component of microwave energy transmission technology, has been a research hotspot.

Currently, most of the research related to rectennas is focused on those below 10 GHz, such as 2.45 GHz [4,5,6] as well as 5.8 GHz [7,8,9], which has been the research hotspot for rectified antennas. For example, a recent paper [10] designed a circularly polarized rectifier antenna operating at 2.48 GHz, which can provide 45% RF–DC conversion efficiency. However, with the increase in antenna gain requirement and the requirement of device miniaturization, it is inevitable that this would push the operating frequency of the antenna from low to high frequency. Compared with the normal microwave band, antennas in the millimeter-wave band [11,12] have the advantages of a lightweight, easy integration with large-scale arrays, and high transmission efficiency. Most of the reports on millimeter-wave rectennas are in Ka-band. For example, the rectenna designed in [13], at 35 GHz, has a rectification efficiency of 35%. In [14], this rectenna has a maximum rectification efficiency of 27.4% at 30 GHz. In [15], a fully metalized alternative to the conventional microstrip patch rectangular antenna is proposed, and it achieves an RF–DC conversion efficiency of 68.5% at 35 GHz. A 4 × 2 microstrip patch rectenna array with a gain of 13.4 dB and an efficiency of more than 80% is proposed in [16]. Compared with Ka-band, both 94 GHz of the W-band and 35 GHz are microwave atmospheric windows with lower transmission loss. For long-distance transmission, the Ka-band rectenna array will face the problem of large array size and weight, compared with this, the miniaturization advantage of the W-band is very obvious. Although the overall rectification efficiency of the W-band rectenna array is not as good as that of the Ka-band, its small size, light weight, easy transportation, and long transmission distance are enough to make it shine in MPT. The results of rectenna research in the W-band are rare, and the dual-band rectifier antenna designed in [17] works at 35/94 GHz with two atmospheric windows and rectification efficiencies of 53% and 37%. A W-band loop on-chip rectenna proposed in [18] has an RF–DC conversion efficiency of only 6.8%. At present, there are fewer research results concerning the W-band rectenna, so it is necessary to study the rectenna of the W-band.

The substrate integrated waveguide (SIW) [19,20,21] technology has been widely used since its invention, and its advantages of low profile, small cost, and light weight make it valuable in the antenna field. In [12], a patch rectenna in Ka-band was designed with the help of the SIW technique with a DC conversion efficiency of 49%. The low profile and easy integration of SIW make it relatively easy to apply to large-scale arrays, just like a 32 × 33 large array SIW back-cavity antenna proposed in [22], with a gain up to 32.88 dB at 93.2 GHz and aperture efficiency of 22.8%. Moreover, the above-mentioned references are the results of the whole rectenna system, the specific performance of the antenna used is not clear, so this paper separately studies the performance of the antenna itself when used as a rectenna, as a supplement to the rectenna system. Thus, with the help of SIW technology, a W-band SIW slot array antenna is proposed in this paper to realize the transition connection between rectifier element and antenna in a small size range, which on the one hand facilitates the miniaturization and planarization of the antenna, and on the other hand facilitates the integration of the antenna for large-scale arrays.

## 2. Antenna Design

**Figure 1:**
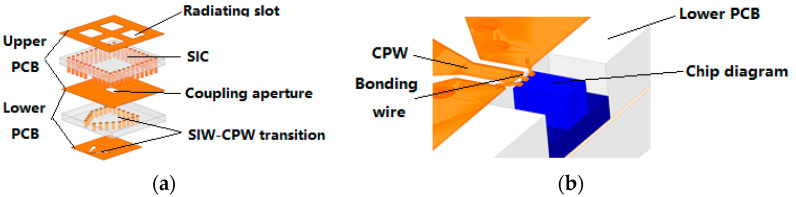
(**a**) Schematic of the rectenna; (**b**) schematic of the transition from rectifier element to rectenna.

Figure 1a is a schematic diagram of the structure of the rectenna, and Figure 1b is a schematic diagram of the connection between the coplanar waveguide at the end of the antenna and the rectifier element, which is proposed in [23]. The coplanar waveguide (CPW) was chosen as the connection structure to the rectifier element because the unique G–W–G structure of the coplanar waveguide corresponds exactly to the pinout of the rectifier element [24]. The antenna receives microwaves from free space through the four radiation windows of the upper substrate integrated resonant cavity (SIC), transmits them to the lower SIW through the coupling gap, then transitions to the coplanar waveguide through the SIW–CPW transition structure, and then transmits them to the rectifier element through the bonding line connected to the rectifier element, converting the microwaves to DC signals and then outputting them. The size of the four parts of the coupling aperture, CPW–SIW transition structure, CPW-rectifier element transition structure, and rectifier element position are controlled so that the whole is less than w = 4.62 mm. Thus, the transition of the rectifier element-antenna is realized in the small size range, and the radiation layer of the antenna is closely arranged at the same time. This can make the antenna easy to be integrated with the large-scale array, and also improves the aperture efficiency of the array. Therefore, to meet the size requirement, some aperture efficiency must be sacrificed, while also maximizing the efficiency within the constrained size.

**Figure 2:**
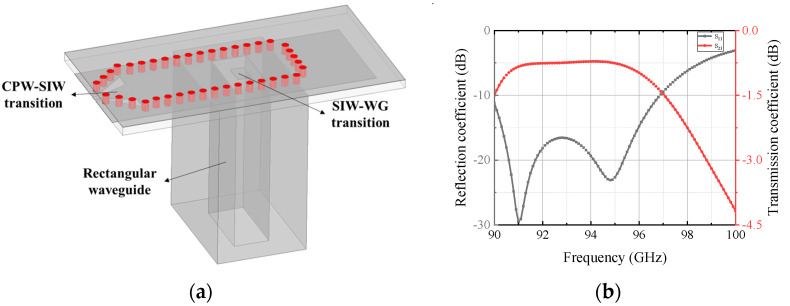
(**a**) Schematic of the CPW–SIW–WG transition; (**b**) simulation results of the S-parameter.

To facilitate the antenna test, a mirror structure of SIW–CPW transition is made at the end of the antenna. The microwave passes through the SIW–CPW–SIW transition structure, and then transitions to the rectangular waveguide, WR10, through the vertical transition structure of the SIW-rectangular waveguide, as shown in Figure 2a. The s-parameters of this structure simulated by HFSS full-wave simulation software are given in Figure 2b. At 90–97 GHz, the reflection coefficient is less than −10 dB and the transmission loss is about 0.78 dB at 92–95 GHz.

**Figure 3:**
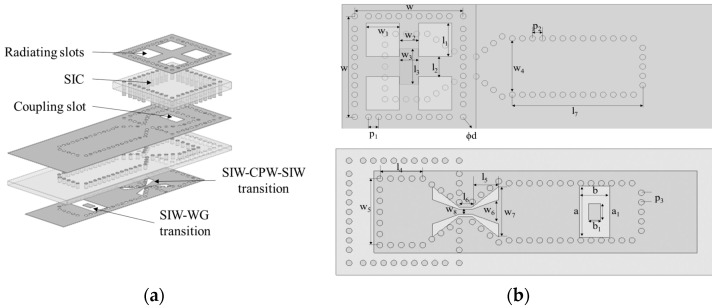
(**a**) Schematic, (**b**) top view and bottom view of the antenna.

**Figure 4:**
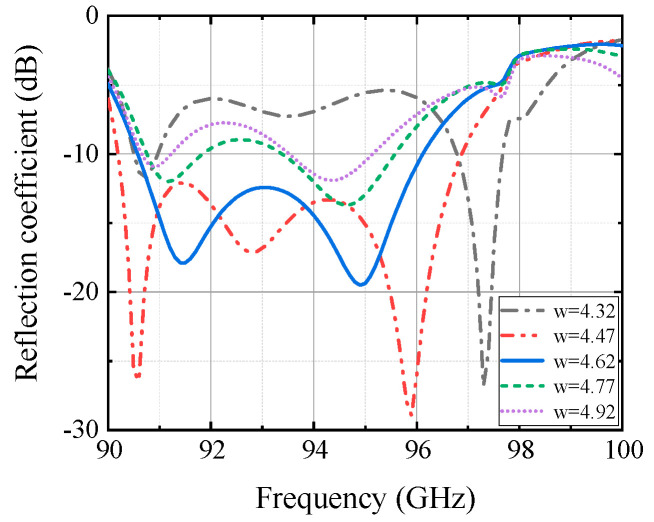
Simulated reflection coefficients of the antenna array at different w values.

**Figure 5:**
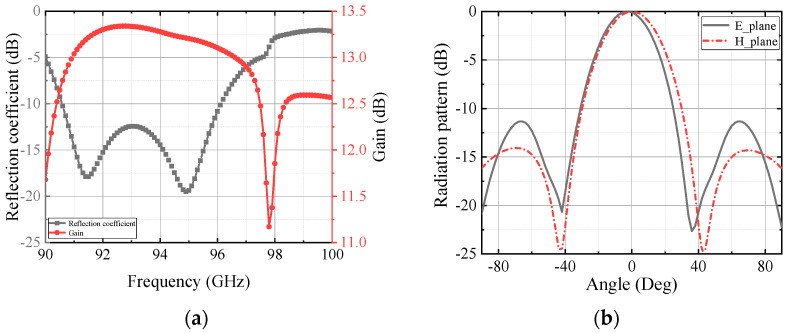
Simulated result: (**a**) reflection coefficient and the gain; (**b**) radiation pattern of the antenna.

The overall structure of the antenna is shown in Figure 3a, the antenna is made of two PCB boards laminated with Rogers PCB/RT Duroid5880 and Rogers PCB/RT Duroid4003 as the substrate material and the thickness is 0.508 mm and 0.3043 mm, respectively. The transmission of this antenna from the feed port to free space is as follows: rectangular waveguide, feed port, rectangular waveguide to SIW transition structure, SIW–CPW–SIW transition structure, SIC coupling aperture, and finally four radiation slots on the uppermost layer. Figure 3b shows the top view and bottom view of the antenna. For the antenna radiation layer, the selected antenna aperture is approximately 1.5λ, through the parameter scan, as shown in Figure 4, to determine the size range of the aperture w of 4.32–4.77 mm, as the subsequent optimization condition, thereby pushing to the rest of the parameters. The width of the antenna feed layer is approximately 1λ, the width w_8_ of the coplanar waveguide is approximately 0.23 mm, in order to connect with the rectifier element, it needs to control its impedance in around 50 Ω, so if both sides of the gap are 0.1 mm, the entire coplanar waveguide width can be controlled in about 0.5 mm. This is to facilitate the subsequent connection with the rectifier element. Finally, by the size range of the determined parameters, the overall optimization of all important parameters is carried out to find out the optimal size of the antenna, and the optimized parameters are shown in Table 1. The reflection coefficient and simulated gain of the antenna are given in Figure 5a. In the 90.5–96 GHz band, the reflection coefficient of the antenna is less than −10 dB, the antenna gain is greater than 13 dB, and the aperture efficiency is greater than 70% by calculation. Figure 5b gives the radiation pattern of the antenna at 94 GHz, the radiation patterns in the E-plane and H-plane maintain good agreement, and the side lobe level is below −11 dB, which proves the good radiation performance of the proposed antenna.

## 3. Measured Results

**Figure 6:**
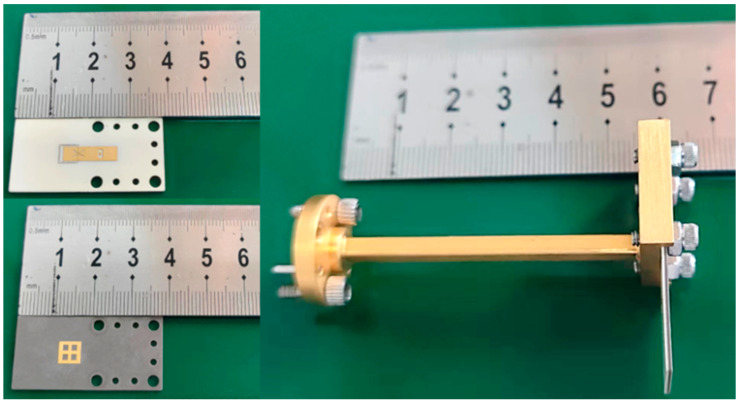
Photograph of the fabricated antenna.

**Figure 7:**
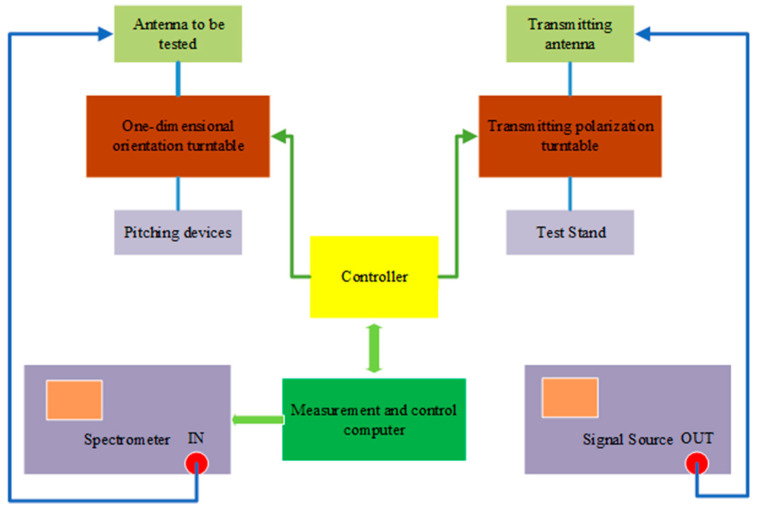
Schematic diagram of the antenna test system.

**Figure 8:**
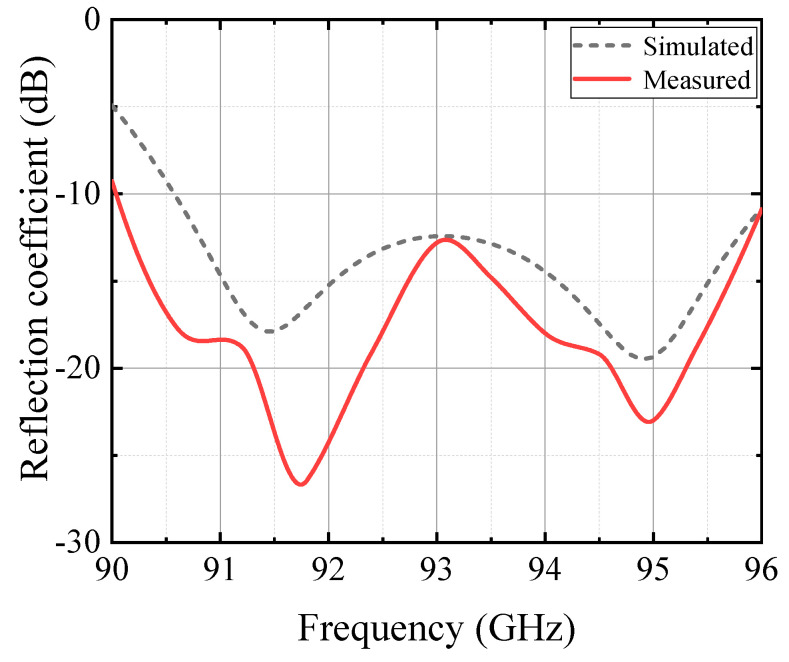
A comparison between the measured and simulated reflection coefficients.

The fabricated antenna is shown in Figure 6. Antenna tests are conducted using the far-field test method, when the antenna is being tested, the transceiver distance should meet the far-field conditions. The test instrument uses a spectrum meter and signal source, the set up method is shown in Figure 7. The measured results of the antenna reflection coefficient compared with the simulated results are shown in Figure 8. At 90–96 GHz, the reflection coefficient is less than −10 dB.

**Figure 9:**
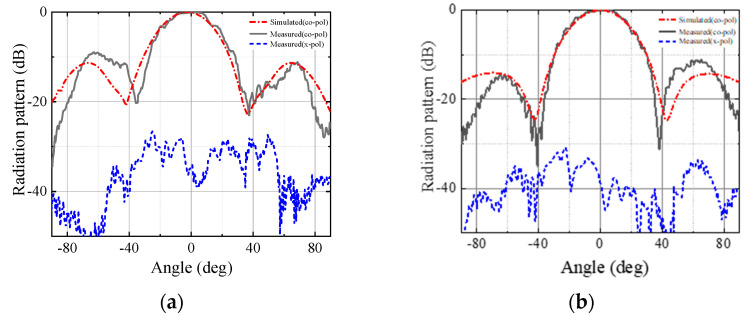
A comparison between the measured and simulated radiation patterns of the antenna at 94 GHz. (**a**) E-plane. (**b**) H-plane.

**Figure 10:**
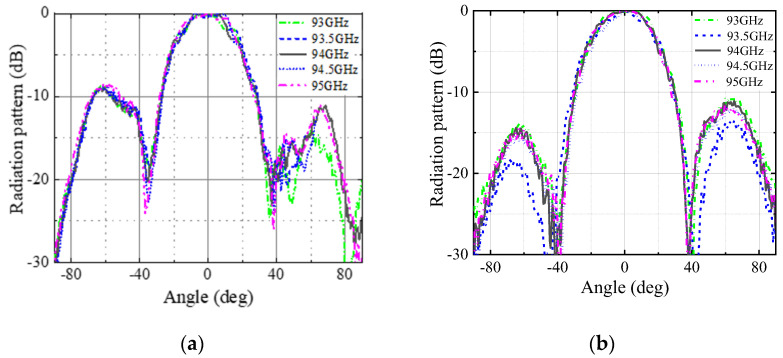
Radiation patterns of the antenna at 93, 93.5, 94, 94.5 and 95 GHz. (**a**) E-plane; (**b**) H-plane.

The simulated and measured radiation patterns of the antenna at the center frequency, 94 GHz, in the E-plane and H-plane are shown in Figure 9. The simulated and measured results are in good agreement. The measured cross-polarization level is −26.2 dB. Figure 10 shows the measured radiation patterns at 93, 93.5, 94, 94.5, and 95 GHz for E-plane and H-plane, respectively. The radiation patterns of the antenna are very consistent at each frequency point.

**Figure 11:**
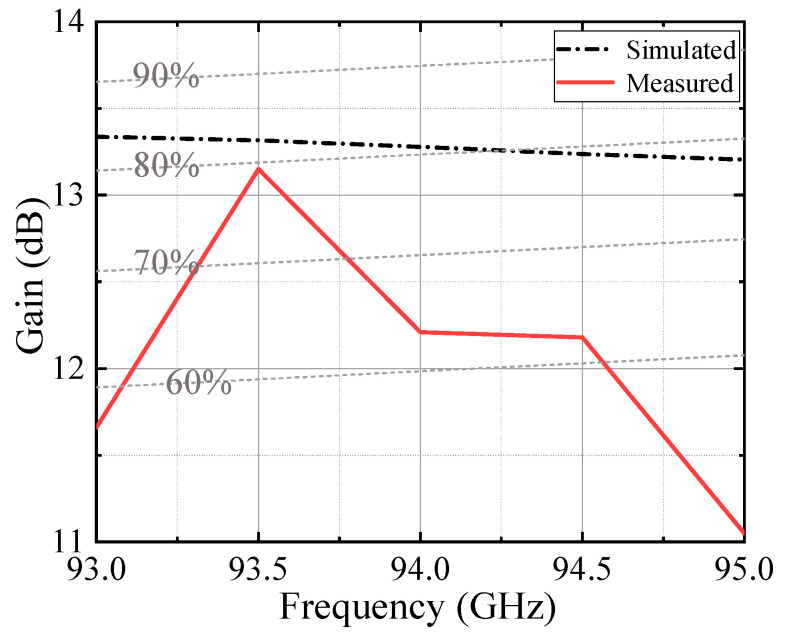
Measured and simulated frequency characteristics of the gain.

Figure 11 shows the measured and simulated frequency characteristics of the gain. The dashed lines indicate the gain at aperture efficiency of 90, 80, 70, and 60%. The gain of the antenna is greater than 12 dB at 93.5–94.5 GHz, and the measured aperture efficiency is higher than 60%. The maximum gain measured is 13.15 dB at 93.5 GHz, and the aperture efficiency is 79%.

The rectenna performance test is given in [23,25], and the rectification efficiency is greater than 20% at 93–94 GHz, and the highest rectification efficiency is 24.3% at 94 GHz.

## 4. Conclusions

In summary, a W-band SIW slot array antenna that can be applied to microwave power transmission systems is proposed in this paper. The coupling aperture, the SIW–CPW transition structure, the CPW-rectifier element transition structure, and the rectifier element are integrated into a small size range, thus allowing the antenna radiating structures to be closely aligned. The close arrangement of the radiation structure improves the aperture efficiency of the antenna, which also facilitates the planarization and miniaturization of the antenna. At 90–96 GHz, the reflection coefficient of the antenna is less than −10 dB, the aperture efficiency is greater than 60% at 93.5–94.5 GHz, and the maximum gain is 13.2 dB at 93.5 GHz, corresponding to the aperture efficiency is 79%. The results prove that the antenna proposed has good performance and can be used in microwave power transmission systems as a rectenna.

## Figures and Tables

**Table 1:** Structural parameters of the antenna.

Parameters	Value	Parameters	Value	Parameters	Value
w (mm)	4.62	w_8_ (mm)	0.23	d (mm)	0.25
w_1_ (mm)	1.52	l_1_ (mm)	1.42	p_1_ (mm)	0.43
w_2_ (mm)	0.94	l_2_ (mm)	0.82	p_2_ (mm)	0.43
w_3_ (mm)	0.79	l_3_ (mm)	1.62	p_3_ (mm)	0.43
w_4_ (mm)	2.33	l_4_ (mm)	1.80	a (mm)	2.54
w_5_ (mm)	3.00	l_5_ (mm)	1.11	b (mm)	1.27
w_6_ (mm)	1.10	l_6_ (mm)	0.56	a_1_ (mm)	0.75
w_7_ (mm)	2.28	l_7_ (mm)	5.39	b_1_ (mm)	0.50

## Data Availability

Not applicable.

## References

[1] Yu F., Yang X.-X. (2022). Progress of Rectenna Arrays for Microwave Power Transmission Systems. Adv. Astronaut. Sci. Technol..

[2] Zhai Z., Jin K., Zhou W., Wang X. A Novel Power Combining Strategy for Rectenna Array of Microwave Power Transmission System. Proceedings of the 2022 IEEE Applied Power Electronics Conference and Exposition (APEC).

[3] Takabayashi N., Kawai K., Mase M., Shinohara N., Mitani T. (2022). Large-Scale Sequentially-Fed Array Antenna Radiating Flat-Top Beam for Microwave Power Transmission to Drones. IEEE J. Microw..

[4] Assogba O., Mbodji A.K., Bréard A., Diallo A.K., Duroc Y. (2022). Tri-Band Rectenna Dedicated to UHF RFID, GSM-1800 and UMTS-2100 Frequency Bands. Sensors.

[5] Dat P.D., Le M.T., Quoc C.N. (2022). Polarization-Insensitive 2.45 GHz Rectenna Using Hybrid Coupler for Wi-Fi Energy Harvesting Application. Meas. Control Autom..

[6] Surender D., Halimi A., Khan T., Talukdar F.A., Antar Y.M.M. (2022). A triple band rectenna for RF energy harvesting in smart city applications. Int. J. Electron..

[7] Saito K., Nishiyama E., Toyoda I. (2022). A 2.45- and 5.8-GHz Dual-Band Stacked Differential Rectenna With High Conversion Efficiency in Low Power Density Environment. IEEE Open J. Antennas Propag..

[8] Takabayashi N., Yang B., Shinohara N., Mitani T. (2022). Lightweight and Compact Rectenna Array with 20W-class Output at C-band for Micro-drone Wireless Charging. IEICE Trans. Electron..

[9] Yi X., Chen Q., Hao S., Chen X. (2022). An efficient 5.8 GHz microwave wireless power transmission system. Int. J. RF Microw. Comput. Aided Eng..

[10] Xu C., Fan Y., Liu X. (2022). A Circularly Polarized Implantable Rectenna for Microwave Wireless Power Transfer. Micromachines.

[11] Wagih M., Hilton G.S., Weddell A.S., Beeby S. (2022). Millimeter-Wave Power Transmission for Compact and Large-Area Wearable IoT Devices Based on a Higher Order Mode Wearable Antenna. IEEE Internet Things J..

[12] Wang Y., Yang X.-X., Tan G.-N., Gao S. (2021). Study on millimeter-wave SIW rectenna and arrays with high conversion efficiency. IEEE Trans. Antennas Propag..

[13] Ren Y.-J., Li M.-Y., Chang K. (2007). 35 GHz rectifying antenna for wireless power transmission. Electron. Lett..

[14] Wei H., Ke W. (2008). Substrate integrated waveguide rectenna array using antipodal linearly tapered slot antenna. Sci. Online.

[15] Chen Q., Liu Z., Cui Y., Cai H., Chen X. (2020). A Metallic Waveguide-Integrated 35-GHz Rectenna With High Conversion Efficiency. IEEE Microw. Wirel. Compon. Lett..

[16] Hoque M.U., Kumar D., Audet Y., Savaria Y. (2022). Design and Analysis of a 35 GHz Rectenna System for Wireless Power Transfer to an Unmanned Air Vehicle. Energies.

[17] Chiou H.-K., Chen I.-S. (2010). High-Efficiency Dual-Band On-Chip Rectenna for 35-and 94-GHz Wireless Power Transmission in 0.13-μm CMOS Technology. IEEE Trans. Microw. Theory Tech..

[18] Shaulov E., Jameson S., Socher E. W-band energy harvesting rectenna array in 65-nm CMOS. Proceedings of the 2017 IEEE MTT-S International Microwave Symposium (IMS).

[19] Esquius-Morote M., Fuchs B., Zürcher J., Mosig J.R. (2013). A Printed Transition for Matching Improvement of SIW Horn Antennas. IEEE Trans. Antennas Propag..

[20] Liu B., Hong W., Kuai Z., Yin X., Luo G., Chen J., Tang H., Wu K. (2009). Substrate Integrated Waveguide (SIW) Monopulse Slot Antenna Array. IEEE Trans. Antennas Propag..

[21] Ma W., Cao W., Tong Y., Zhang B. (2022). Sidelobe Suppression for Wideband Slotted SIW Cavity-Backed Antenna Array. IEICE Electron. Express.

[22] Wu Y.W., Hao Z.C., Miao Z.W. (2020). A Planar W-Band Large-Scale High-Gain Substrate-Integrated Waveguide Slot Array. IEEE Trans. Antennas Propag..

[23] Liu L., Yu C., Meng F., Zheng Q. W-Band rectenna array based on SIW slot antenna. Proceedings of the 2020 International Conference on Microwave and Millimeter Wave Technology (ICMMT).

[24] Kazemi R., Fathy A.E., Yang S., Sadeghzadeh R.A. Development of an ultra wide band GCPW to SIW transition. Proceedings of the 2012 IEEE Radio and Wireless Symposium.

[25] He P., Zhao D., Liu L., Xu J., Zheng Q., Yu C., You X. (2021). A W-Band 2 × 2 Rectenna Array With On-Chip CMOS Switching Rectifier and On-PCB Tapered Slot Antenna for Wireless Power Transfer. IEEE Trans. Microw. Theory Tech..

